# Further Observations on the Effect of C. parvum and Anti-Tumour Globulin on Syngeneically Transplanted Mouse Tumours

**DOI:** 10.1038/bjc.1972.11

**Published:** 1972-04

**Authors:** M. F. A. Woodruff, M. P. Inchley, Noreen Dunbar

## Abstract

The inhibitory effect of an i.v. or i.p. injection of *C. parvum* on intrastrain transplants of a mammary carcinoma in A/HeJ mice has been confirmed, and it has been shown further that *C. parvum* inhibits the growth of transplants of sarcomata induced with methylcholanthrene both in this strain (members of which lack the fifth component of complement) and in CBA mice (which are not complement deficient). In experiments with the mammary carcinoma, 2 injections of *C. parvum* on days + 3 and + 9 were more effective than a single injection on day + 3; injections on days + 3 and + 6, or + 3 and + 12, appeared to be marginally less effective than on days + 3 and + 9, but the difference was not statistically significant.

Development of the CBA sarcoma was inhibited to about the same extent if, instead of treating the mouse with *C. parvum,* the tumour cells were pre-incubated with anti-tumour globulin (ATG) in the absence of complement prior to inoculation, and the effect of combining these procedures was much greater than that of either alone. Pre-incubation with ATG had a similar but less marked effect on the development of the mammary carcinoma but had no effect on the A/HeJ sarcoma. Injection (i.v.) of ATG did not inhibit the growth of any of the tumours in these experiments and possible reasons for this are discussed.


					
Br. J. Cancer (1972) 26, 67

FURTHER OBSERVATIONS ON THE EFFECT OF C. PARVUM

AND ANTI-TUMOUR GLOBULIN ON SYNGENEICALLY

TRANSPLANTED MOUSE TUMOURS

M. F. A. WsOODRUFF, Mr. P. INCHLEY AND NOREEN DUNBAR

Fronm the Department of Surgery, University of Edinburgh

Receive(d foi publication December, 1971

Summary.-The inhibitory effect of an i.v. or i.p. injection of C. parvum on intrastrain
transplants of a mammary carcinoma in A/HeJ mice has been confirmed, and it has
been shown further that C. parvum inhibits the growth of transplants of sarcomata
induced with methylcholanthrene both in this strain (members of which lack the
fifth component of complement) and in CBA mice (which are not complement
deficient). In experiments with the mammary carcinoma, 2 injections of C. parvum
on days + 3 and + 9 were more effective than a single injection on day + 3; injections
on days + 3 and + 6, or + 3 and + 12, appeared to be marginally less effective than
on days + 3 and + 9, but the difference was not statistically significant.

Development of the CBA sarcoma was inhibited to about the same extent if,
instead of treating the mouse with C. parvum, the tumour cells were pre-incubated
with anti-tumour globulin (ATG) in the absence of complement prior to inoculation,
and the effect of combining these procedures was much greater than that of either
alone. Pre-incubation with ATG had a similar but less marked effect on the develop-
ment of the mammary carcinoma but had no effect on the A/HeJ sarcoma. Injection
(i.v.) of ATG did not inhibit the growth of any of the tumours in these experiments
and possible reasons for this are discussed.

As already reported, the growth of
intrastrain transplants of a mammary
carcinoma in A-strain mice may be inhibi-
ted to a moderate extent by treating the
recipient with a single intravenous or
intraperitoneal injection of an appro-
priate strain of C. parvum (Woodruff
and Boak, 1966; Smith and Woodruff,
1968; Woodruff and Inchley, 1971a) or
by incubating the tumour cells prior to
inoculation with heterospecific antitumour
globulin (ATG) (Woodruff and Smith,
1970). Significantly greater inhibition
may be obtained by combining these
procedures (Woodruff and Inchley, 197 la).

The experiments now reported extend
this work in two directions. In the first
place we have studied the effect of
treating the animal with ATG, either
alone or in combination with C. parvum.
Secondly, in order to determine whether
or not the results obtained previously
were peculiar to the particular model
chosen, in which the tumour was of viral

6

origin and the mice lacked the fifth
component of complement (C5), we have
set up similar experiments with 2 fibro-
sarcomata induced with methylcholan-
threne, one in A/HeJ mice (i.e. the strain
used in previous work with the mammary
carcinoma) and one in CBA mice, which
possess all components of the complement
system.

MATERIALS AND METHODS

Six experiments were performed, 2 with
A/HeJ mammary carcinomata, 2 with a
fibrosarcoma induced in A/HeJ mice with
methyleholanthrene and 2 with a fibrosarcoma
induced in CBA mice by the same carcino-
gen. The detailed protocols are shown in
Tables I, II and III. It should be noted that
in these experiments C. parvum was injected
on day + 3 (i.e. 3 days after tumour inocula-
tion) and not on day -2 as previously.

Mice.-The mice were adult (18-26 g)
females of strain A/HeJ or CBA/H. The
A/HeJ mice were obtained from the Jackson
Memorial Laboratories, Bar Harbor, Maine,

M. F. A. WOODRUFF, M. P. INCHLEY AND NOREEN DUNBAR

0 0 100mnf 100

10     C' 4  CO Co

4      CO t  X

OW  CO E.

C)

6D  C >
_ -

0 COO .O

C)   CO
a; i " 0   10

(?   m O  ^-

CC)C

la _ O
C O _00
14E

C)  C 0

1p 0010-

0
E--

._ ._ ._ .

;tt

4 _M

ED    ., 4

pi   --4

C)

a2 to o0   i 40    0
4.C) O. COCO       CO

0 0000              0

01
CO

CCO4

r  CD
1-

- CO
O ^

-CO

- C
01  -

1. 0

01 01

CO4

6 re
0 CO

- t-

O o

-0
-0

4

,

^

o 0

10 CO

10

Ci o

10  -  o~  o~

+ +

+ .?

.4. .4.>
QO w bO w

grg---

10 10 ZZ ZZ

ACO ;,CO AC

._ +  *_ +  _ +

0   00   00
_   0-0  0-0

4-D  z

C a 3   C a  Ca  Ca  C)

-Q-a  a4 4  4-D4 6

0           4) )IC) D
P-1  t7l h -t)  4  f. In

0 --

60   4 01t CO10

14

z*

04

rz.

co

66

*~ .~

C) C)

1414

4. _I ,

) D

k f-4

P-4 P-4

CO   C    inCO    =10

o     Z

4-'   0

0    .
o -4

4   o

.  0  P.,

C) o C
,    W; o

-  0t 0. 15        Q t

C>~~~~~~C

00     -

*~~~~~~~~~~4 *  ;;

CO b         .4

o0 CO         -     O

-  10     C:COfD
Co       lo  ?    C )

o         0^  o  X  t t:

-, 04 10+ -~

~~~~~~~~~ -~~~~~~~~~~~~4-

C)4X- w

o 0    >

CO 0)    a

?  +   +   ?, V

tC .C .C oCo .o
-  o      oi"oi"t  8H0i

P-  H    i  r-  C;-4

14 b

0  0   0  0    P    a

&.c     ;.  & t t  tl ;4

4a  4a  4--  -4n   -

o  C)  C)  C  C         4 C

C -; t-3

CO *O CO C CO

- 01  CO  04  100

.0 0
o            *~~~~~~~~~~~~~~4

C)             ~~~ ~~~~4.  03
C)4

CO~~~~~~~~0

68

~q

*l~ ,i
ct

.,
* ca

E-1

_

m0 "-
9 'O

C)

C)

C)

0O
C)
C)
14a

w

E-q

4-
C)
C)

E

I

5

114

3

? -.4

. -4

?2;

IriJ } -d )-   1-   i-J  -d -  H  1-H

EFFECT OF C. PARVUM AND ANTI-TUMOUR GLOBULIN ON MOUSE TUMOURS 69

U.S.A.; the CBA mice were bred in c
laboratory from a strain obtained origina
from the MRC Laboratory Animals Cent
Carshalton, Surrey.

Tumours. The mammary carcinoma
originated spontaneously in old fem.
A/HeJ mice. The sarcomata were induc
by giving a single intramuscular injection
0-5 mg methylcholanthrene in 041 ml trib
tanoin to A/HeJ or CBA female mice ag
8-10 weeks. The tumours were propagat
routinely by subcutaneous (s.c.) transplan
tion of a small piece of tissue, but in t
experiments transplantation was perform
by s.c. injection of cell suspensions prepar
with pronase as described previou:
(Woodruff and Boak, 1966). The proporti
of viable cells, determined by a dye-exclusi
test with trypan blue, ranged from 80

9000. The tumour dose is expressed as t
number of viable cells.

The 2 mammary carcinomata had bc
been transplanted once, before being used
the experiment. Both sarcomata had be

. fi

1

E

ck 1

w

LI-

FD
oz

:u
2

transplanted 4 times before being used for
the first time experimentally.

C. parv m-.A formalin-killed suspension,
Batch EZ174, obtained from the Wellcome
Foundation by courtesy of Dr J. Cameron,
was used throughout. This is the same as the
first of the 2 preparations used in our last
experiment (Woodruff and Inchley, 1971a).

ATG.-ATG (A/HeJ-Ca) was prepared
by salt precipitation and batch chromato-
graphy from the serum of rabbits immunized
with a mixture of tumour cells prepared
with pronase from several different A/HeJ
mammary tumours according to the schedule
described previously (Woodruff and Inchley,
1971a).

ATG (A/HeJ-Sa) and ATG (CBA-Sa)
were prepared similarly from the serum of
rabbits immunized in the same manner
with  cells prepared  with pronase from
transplants of one or other of the fibro-
sarcomata used in the experiments. The
cytotoxic titres-1 of these preparations
by the techniques previously described

0000 ,
000,
000
.00
.0-1
.000,
.00
.00

..10
.00,

1.41                                                                 .00, .00
1000,
1-1

.00,

1000,                                                  .010
I.,

O.-,
.01
.01,

..*, 4arl,
.00,
.01,

24         29                      41                                 57
DAYS AFTER INOCULATION WITH TUMOUR CELLS

FiG. 1. Effect of initial cell (lose, pre-incubation of tumour cells with ATG and injection of C. parvum,

on the growth of mammary tumour transplants in A/HeJ mice. First experiment.  O   D 10 6
viable tumour cells s.c. No treatment. A----A 105 viable tumour cells s.c. No treatment.
0--- 0 104 viable tumour cells s.c. No treatment.    *     *  106 viable tumour cells s.c.
C. parvum i.p. day + 3.  *     * 106 viable tumour cells s.c. Cells were pre-incubated with
ATG. * * 106 viable tumour cells s.c. Cells were pre-incubated with ATG. C. parvum
i.p. day + 3.

M. F. A. WOODRUFF, M. P. INCHLEY AND NOREEN DUNBAR

0

4 0

.O =

4-  IC M =n CD =O  CD  CD  CD CD  CD CD
0   0;

t; - -W

B w

0   C  E  .   .   .   .

4t0  0   g  W m CD   =

: --- -

_ O _O -

- --^^-

b _0 _1

0  sc

WH   ^

M  - . ld

+

,\ O O O O       O

11: _4 _4 _- _    _

N- CN m0 cO0;i
c4   -4    iOo      c0P-

-   -o

-  CO -

- - - -

-  -O ~ 0

- -  - o]

? .+
m
+

M.0 ZZZ

0 00 00

i <,,    ~~~~~~~~~~~~E-- E-  $
+', ? ~   -4-  +'C  =  gQ t

00  00
0 8

o  ss  sH    Hs

0

$14  -1  O' 4  10i  I

0

~14~

co    r-        o

r..

0   r

o ,o

oa  0

00 0O t4      00 4 Ci  O

14-)

04.4
W 0

cio~~~~~~  0~It

"0

rz C~i  te  e 6 E-i.

.  *   *   *  -   *  -   w. o

141   4L

~~4C0~~

-.4  4 ~ ~ ~ 0  .

00O  0   00O  0 0

~   40
00  0  00  00~~~~~~~~~4-

0

oH   o  ooo          t W

0 0
-4-) 4 o  CtO~ 4 oo) 4-?) C4

0 00 _I  00 O O   0

. ~~~~~~>H

CX~~~~~~~~~~~~~~~~~~~~~~I o  o o

~~~~~~~~P.1 -

70

01
Gq

OD

W

* 14 -

0t

0

0

0
.1

C-

0

14

EH

M

4

C)
0

0 -

I

- Go

--4 I.-O ?-4 aq ;:? -
-4 -4 -4

z x x :? ?;

EFFECT OF C. PARVUM AND ANTI-TUMOUR GLOBULIN ON MOUSE TUMOURS 71

(Woodruff and Smith, 1970), using guinea-pig
C and taking 50% cell death as the end point,
were 64, 32 and 64 respectively. Normal
rabbit IgG (NRG) was prepared from the
pooled serum of normal rabbits.

Pre-incubation of tumour cells.-2. 107 cells
were incubated in 2-6 ml Dulbecco's solution
containing 6-5 mg of the corresponding ATG
for 60 min at 370C without added comple-
ment.

RESULTS

The results are shown in the tables
and figures. They may be summarized
as follows:

A/HeJ mammary carcinoma

In the first experiment (Table I;
Fig. 1) a single i.p. injection of C. parvum
on day + 3 appeared to be at least as
effective in inhibiting tumour growth as an
injection on day - 2 had been in the

_f%

E

w

I-

w

a
n

0

z
w
.M

previously reported experiments. Pre-
incubation of tumour cells with ATG,
though not with NRG, resulted in moder-
ate inhibition of tumour growth. Com-
bination of both these procedures caused
marked inhibition initially but after about
5 weeks the mean tumour diameter in
this group of mice did not differ signifi-
cantly from that of C. parvum treated
mice which received non-incubated cells.
Intravenous injection of ATG alone had
no demonstrable effect in this experiment,
and if anything it appeared to reduce the
inhibitory effect of a subsequent i.p.
injection of C. parvum.

In the second experiment (Table I)
the tumour grew unusually quickly in the
control mice and the inhibitory effect of a
single i.p. injection of C. parvum was
correspondingly small; 2 doses, however,
had quite a marked effect. Administra-

, 0'

/

/

Pr/

DAYS AFTER INOCULATION WITH TUMOUR CELLS

FIG. 2.-Effect of initial cell dose and injection of C. parvum on the growth of sarcoma transplants

in A/HeJ mice. First experiment.     L1----J 104 viable tumour cells s.c.   No treatment.
A---- A   103 viable tumour cells s.c. No treatment.  O----      102 viable tumour cells s.c.
No treatment.  *      * 104 viable tumour cells s.c. C. parvum i.p. day + 3.

F%

2M. F. A. WOODRUFF, M. P. INCHLEY AND NOREEN DUNBAR

tion of the second dose on day + 9
appeared to be marginally more effective
than on day + 6 or + 12 but the differ-
ence is not statistically significant.
A/HeJ sarcoma

In the first experiment (Table II;
Fig. 2) the tumour grew quite rapidly
even after inoculation of only 100 viable
cells. A single i.p. injection of C. parvum
on day + 3 caused marked inhibition of
growth. Neither pre-incubation of the
tumour cells with ATG nor i.v. injection of
ATG alone had any demonstrable effect,
nor did these procedures augment the
effect of a subsequent injection of C.
parvum; indeed i.v. injection of ATG
appeared if anything to reduce this.

The second experiment (Table II;
Fig. 3) provided further evidence of the
powerful inhibitory effect of a single

,i-

16-

14-
E.
E

o~ 12-
w

4-

2 10-

0

w 8 -
0

1-. 6 -

z   SC

2-

injection of C. parvum, and the results
suggest that intravenous injection is
even more effective than intraperitoneal.
CBA/H sarcoma

In the first experiment (Table III;
Fig. 4) growth of the tumour was inhibited
to about the same extent by pre-incubation
of the cells with ATG or by a single i.p.
injection of C. parvum. Pre-incubation
of the tumour cells with ATG combined
with injection of C. parvum    was still
more effective. Intravenous injection of
C. parvum seemed initially to be more
effective than i.p. injection but after a
few weeks the difference was no longer
apparent.

In the second experiment (Table III;
Fig. 5) the inhibitory effect of C. parvum
was confirmed, but i.p. injection was no
less effective than i.v. As in the experi-

.13

-. 1-.1 -l  -
---o

~~~~A  - ~~~0, -I  o

A .--

16

I             I l

22            28                35

DAYS AFTER INOCULATION WITH TUMOUR CELLS

42

FIG. 3.-Effect of initial cell dose and injection of C. parvum on the growth of sarcoma transplants

in A/HeJ mice. Second experiment.     O  -  ] 104 viable tumour cells s.C. No treatment.
*     * 104 viable tumour cells s.c. C. parvum i.p. day + 3.  x  x 104 viable tumour cells
s.c. C. parvum i.v. day + 3. A ----A 103 viable tumour cells s.c. No treatment. * A
103 viable tumour cells s.c. C. parvum i.p. day + 3.  O -   102 viable tumour cells s.c. No
treatment.  *     * 102 viable tumour cells s.c. C. parvum i.p. day + 3.

I

I

72

_0

vJ

1.11

EFFECT OF C. PARVUM AND ANTI-TUMOUR GLOBULIN ON MOUSE TUMOURS 73

O  oS0 01 E  o

0~~~~~~~~~~~~0
4) C O   -.

*-CO  _

N --  -

o e     0  -  Xjc~  ir~  N  CO
St  P E -  ? -  m?  e -

04~~

;~~~ -   O0  _~  _O  _Not

00  00

0O  - t  -  o   _ ^   C e o ^  C O  _

4--

es ~ ~~  01  - _ 0_-  -  _ 0_1

Ca1     N COO  0 -

;  ?   O   0  0  01  - _   '_ 1  -

.N "e+  s

t~~~~C O1 -X  01 XJ  1O  m

~s":  t'01     - d _O -^ C

a ~ ~ ~ ~ -  CO O  4  o i^O ^o

X    CD ~o  o  c&  4  o' c  4
o                c

Ca ~ ~ ~ ~ ~ ~ C

0 :;   ? ~ CO~CO zC

+*~ +b  +  ?

W          ;  _  _ _ _ _ _ _ _~~~~~~~01"

<   W  Ztt  tZ6Z t*tZ  *
*S~~~~~~i U

Ct ~ 0  0  0  0 0

a ~ ~ ~ ~ " 2~  " c  s p  o p

^ U t C CVCe

. '.4p (3)

;4
4) &4 .4.

k 0 0

N       ?t)

C4..4*
0 a)

6 .0   00

?', E

?14
O

0 -4

k
0

4'?,

04

X 4---)

rA m

r-4

PLI

X      01     r      CD    1

~o CS    00     4      l4'

_      _4    _-     _4     _

-  1

0   CO  0
010 - 01

CO 01 0

_O  0   CO
O   0   10
~C O

,I

Ca

>1

CO  CO    COC     CO  Na     D   CO

t,-, *  ;_,  0    0,   ;,

00   Co   00 0    a(   r-   00   0

.   O

CO N
0    -

CO
- C

0

.-4

ca

O
0

._

0
0

C-

CC

0
C0
z

cd

+     e;l +

?   + 0

bl d  bD) m  bD  d

Z   Z  10   01 to

0
6.m~~~~~~~~~~~~~.

fH

CD e   e   p    0
;. O.  t   O   O

0
CO
C..
-   t  CO D    1    0

-~~~~~~~~~~~   H~~~~~~~~-

0                   * D
0;  ;   ;  X   4
cdc0    d  d   c
CO  O   s  s   s

M. F. A. WOODRUFF, M. P. INCHLEY AND NOREEN DUNBAR

ments with the A/HeJ sarcoma, however,
injection of ATG did not inhibit growth
of the tumour and appeared to reduce the
inhibitory effect of a subsequent dose of
C. parvu m.

DISCUSSION

It seems clear that the inhibitory
effect of C. parvum is not peculiar to the
model previously investigated but extends
to intra-strain transplants of chemically
induced sarcomata in both complement
(C5) deficient and non-complement defi-
cient mice. The effect of C. parvum on
cholanthrene-induced sarcomata in CBA
mice has also been studied by Currie and
Bagshawe (1970). The inhibition of tumour
growth in animals treated with C. parvum
alone appears to have been weak in com-
parison with that obtained in the present
experiment, probably because of differen-

E

ck:
w

w

D
0

LLJ

x

I.J

ces in the strain of organism and in the
method of preparing the vaccine, which
was heat-killed  (as against formalin-
killed). It is noteworthy, however, that
despite this, Currie and Bagshawe observed
a marked synergistic effect when a single
injection of C. parvum was preceded by a
course of injections of cyclophosphamide.

The effect of pre-incubating the tumour
cells in the present experiments with
heterospecific ATG was variable, inhibi-
tion of growth being greatest with the
(CBA sarcoma but clearly demonstrable
also with the A-strain mammarv car-
cinoma. The synergistic effect of com-
bining pre-incubation of the cells and
injection of C. parvumr, which was so
marked in previous experiments with the
A-strain mammary carcinoma, was less
striking in the present experiment with
this tumour, possibly because of the

-

DAYS AFTER INOCULATION WITH TUMOUR CELLS

Fie1. 4. Effect of initial cell dlose, pre-incubation of ttumour cells with ATG, and( injection of C.

p(teurn, on the growth of sarcoma transplants in CBA/H mice. First experiment.  DH -      104
viable tuimour cells s.c. No treatment. A---- A 103 viable tumour cells s.c. No treatment.
0---- 0   102 viable tumour cells s.c. No treatment.   *     *  104 viable tumour cells s.c.
C. por?lum i.p. clay + 3.  x -   x 104 viable tumour cells s.c. C. parvum i.v. day + 3. A  A
104 viable tumour cells s.c. Cells were pre-incubated with ATG.  *     * 104 viable tumour
cells s.c. Cells were pre-incubated with ATG. C. parvum i.p. day + 3.

74

ni

1-1
I.,

,.-IO,
oll

EFFECT OF C. PARVUM AND ANTI-TUMOUR GLOBULIN ON MOUSE TUMOURS

F

LLJ

w

llJ
w

.M
4
5

D
0
:D
z
uJ

14      17         21         25      28
DAYS AFTER INOCULATION WITH TUMOUR CELLS

FIG. 5. Effect of injection of C. parvum and ATG on the growth of sarcoma transplants in CBA/H

mice. Second experiment. All the mice received 104 viable cells s.c.  L--- OL No treatment.
*     * C.parvumi.p.day + 3. *      *ATG5mgi.v.day + 1, + 2. *         0 ATG5mg
i.v. day + 1, + 2. C. parvum i.p. day + 3. x  x ATG 5 mg i.v. day + 1, + 2. C. parvum
i.v. day + 3.

different timing of the C. parvum injection,
but was quite dramatic with the CBA/H
sarcoma.

The observation that i.v. injection of
ATG did not inhibit the growth of any
of the tumours studied is in line with the
experience of other investigators (see
Motta, 1971, for review), who have found
that, with a few exceptions, treatment
with antiserum is ineffective with mouse
tumours other than leukoses.

In discussing possible reasons for the
failure of passive immunotherapy it is
convenient to begin by considering the
factors which determine the susceptibility
of cells to destruction consequent on
exposure to antibody and complement.
These include intrinsic properties of the
cells, the specificity and class of antibody,
the availability of necessary complement
components, and the milieu in which the
reaction occurs.

In the case of lysis of both normal and
neoplastic nucleated cells by isoantibody
in vitro, the concentration and distribu-
tion of corresponding antigenic determin-

ants on the surface of the cell are of crucial
importance (Moller and M6ller, 1962;
Winn, 1965), though other properties of
the cell, such as the capacity to repair
lesions resulting from activation of com-
plement on the cell surface, may also be
relevant. Lysis of nucleated cells by
heterospecific antibody in vitro has not
been extensively analysed, but it might
be expected that the number of combining
sites would be greater than with isoanti-
bodies and in consequence less likely to be
a limiting factor, and results obtained in
this laboratory (Woodruff and Inchley,
197 lb) with one particular system are
consistent with this suggestion. Whether
or not this holds good generally, however,
it seems clear that the failure of passive
immunization in the present experiments
cannot be attributed wholly to some
intrinsic property of either the tumour
cells or the antibody, since, when sensi-
tized with the corresponding antibody,
the cells of all the tumours were lysed in
vitro by guinea-pig complement, and, as
mentioned above, those of two of them

75

76       M. F. A. WOODRUFF, M. P. INCHLEY AND NOREEN DUNBAR

proved to be at a disadvantage as com-
pared with normal cells when injected in
vivo.

It seems likely that the explanation is
to be found in the amount of antibody
reaching the tumour cells under the
conditions of the experiments, and on the
availability of complement. To investigate
this we are studying the uptake of radio-
actively labelled antibody by tumours
in vivo and the complementary activity
of serum from tumour bearing animals.
Another approach is to try to prepare
antibody which is more highly tumour
specific, and therefore less liable to be
mopped up by normal tissues, and to
study the effect of injecting this alone
or with exogenous complement.

Another possibility, which may also
be tested by using sera which are more
tumour specific, is that the ATG used in
the present experiments exerted an
immunosuppressive effect which was
sufficient to counterbalance any damaging
effect it had on the tumour.

Various agents which stimulate
immunological responsiveness in a non-
specific way, including Bacillus Calmette
Guerin (BCG) and synthetic polynucleo-
tides, have been tried clinically in patients
with acute lymphoblastic leukaemia by
Mathe (Mathe et al., 1968; Mathe et al.,
1970; Mathe, 1970) and some of the results
have been encouraging. In our hands
(Woodruff and Dunbar, unpublished) these
have proved much less effective against
mouse tumours than the preparation of
C. parvum used in the present experi-
ments. There would seem therefore to
be a good case for undertaking a prelimin-
ary clinical trial of this preparation, either
alone or in combination with chemo-
therapy or some other form of immuno-
therapy, in selected patients with residual
cancer, provided that the criteria of
uniformity and safety applicable to bacter-
ial vaccines can be met.

We gratefully acknowledge a generous
gift of C. parvum from the Wellcome
Foundation (Dr J. Cameron). We are
indebted also to Mrs Evelyn Pawley
for skilled technical assistance.

The work was supported by a grant
from the Medical Research Council.

REFERENCES

CURRIE, G. A. & BAGSHAWE, K. D. (1970) Active

Immunotherapy with Corynebacterium parvum
and Chemotherapy in Murine fibrosarcomas.
Br. med. J., i, 541.

MATHA, G. (1970) Immunological Treatment of

Leukaemias. Br. med. J., iv, 487.

MATHAl, G., AMIEL, J. L., SCHWARZENBERG, L.,

SCHNEIDER, M., CATTAN, A., SCHLUMBERGER, J. R.,
HAYAT, M. & DE VASSAL, F. (1968) Demonstra-
tion de l'Efficacite de l'Immunotherapie Active
dans la Leuc6mie Aigue Lymphoblastique
Humaine. Revue fr. Etud. clin. biol., 13, 454.

MATH1, G., AMIEL, J. L., SCHWARZENBERG, L.,

SCHNEIDER, M., HAYAT, M., DE VASSAL, F.,
JASMIN, C., ROSENFELD, C., SAKOUHI, M. &
CHOAY, J. (1970) Remission Induction with
Poly IC in Patients with Acute Lymphoblastic
Leukaemia (Preliminary Results). Rev. fr. Etudes
clin. biol., 15, 671.

MOLLER, E. & MOLLER, G. (1962) Quantitative

Studies of the Sensitivity of Normal and Neo-
plastic Mouse Cells to the Cytotoxic Action of
Isoantibodies. J. exp. Med., 115, 527.

MOTTA, R. (1971) Passive Immunotherapy of

Leukaemia and Other Cancer. Adv. Cancer Res.,
14, 161.

SMITH, LINDSAY H. & WOODRUFF, M. F. A. (1968)

Comparative Effect of Two Strains of C. parvum
on Phagocytic Activity and Tumour Growth.
Nature, Lond., 219, 197.

WINN, H. J. (1965) Effect of Complement on Sensi-

tized Nucleated Cells. In Complement, (Ciba
Foundation Symposium). Ed. G. E. W.
Wolstenholme and Julie Knight. London:
Churchill. p. 133.

WOODRUFF, M. F. A. & BOAK, J. L. (1966) Inhibitory

Effect of Injection of C. parvum on the Growth
of Tumour Transplants in Isogeneic Hosts. Br.
J. Cancer, 20, 345.

WOODRUFF, M. F. A. & INCHLEY, M. P. (1971a)

Synergistic Inhibition of Mammary Carcinoma
Transplants in A-strain Mice by Antitumour
Globulin and C. parvum. Br. J. Cancer, 25,
584.

WOODRUFF, M. F. A. & INCHLEY, M. P. (1971b)

Cytolytic Efficiency of Rabbit-anti-mouse Anti-
lymphocytic Globulin and its Augmentation by
Antiglobulin. Clin. exp. Immun., 9, 839.

WOODRUFF, M. F. A. & SMITH, LINDSAY H.-(1970)

Cytotoxic Efficiency and Effect on Tumour
Growth of Heterospecific Antilymphocytic and
Antitumour Sera. Nature, Lond., 225, 377.

				


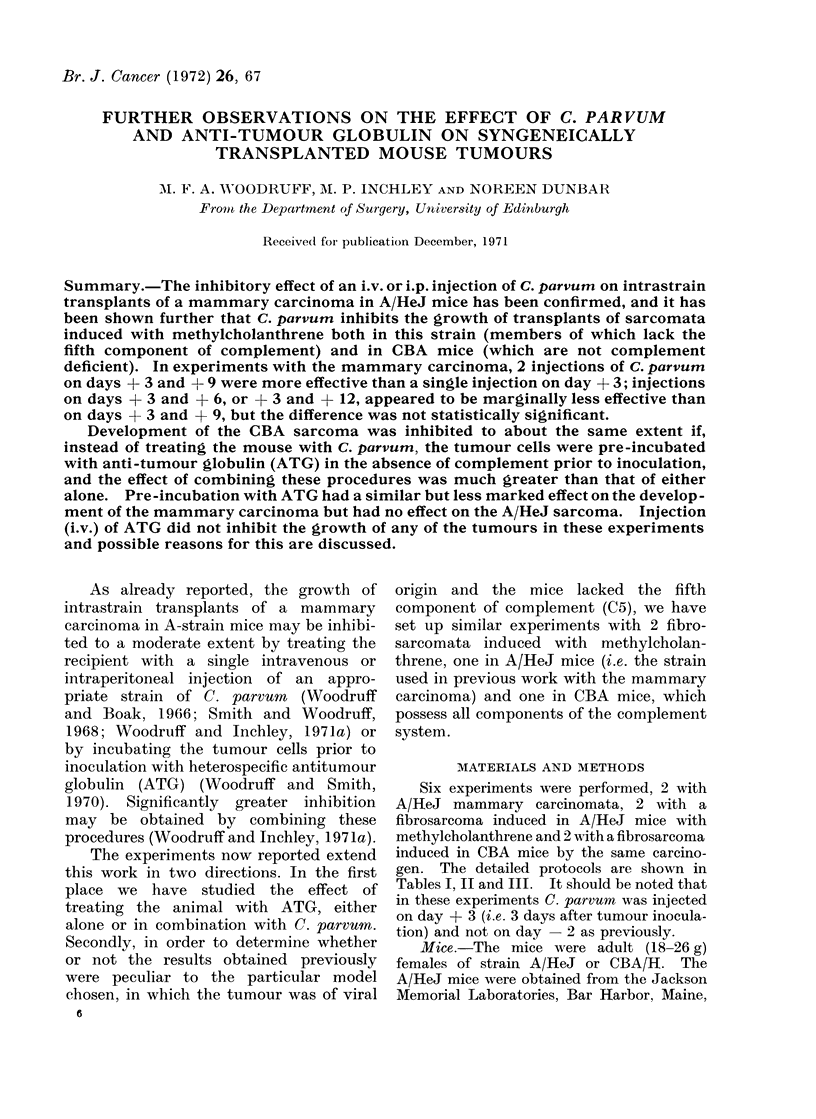

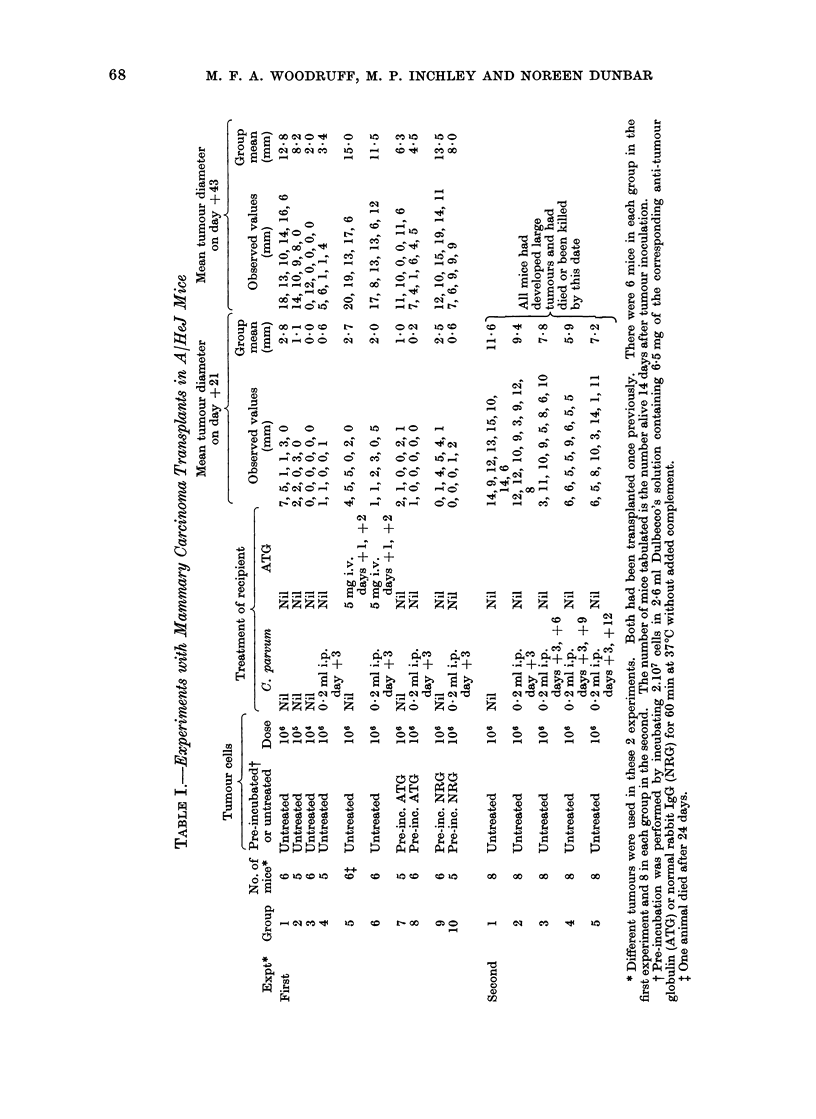

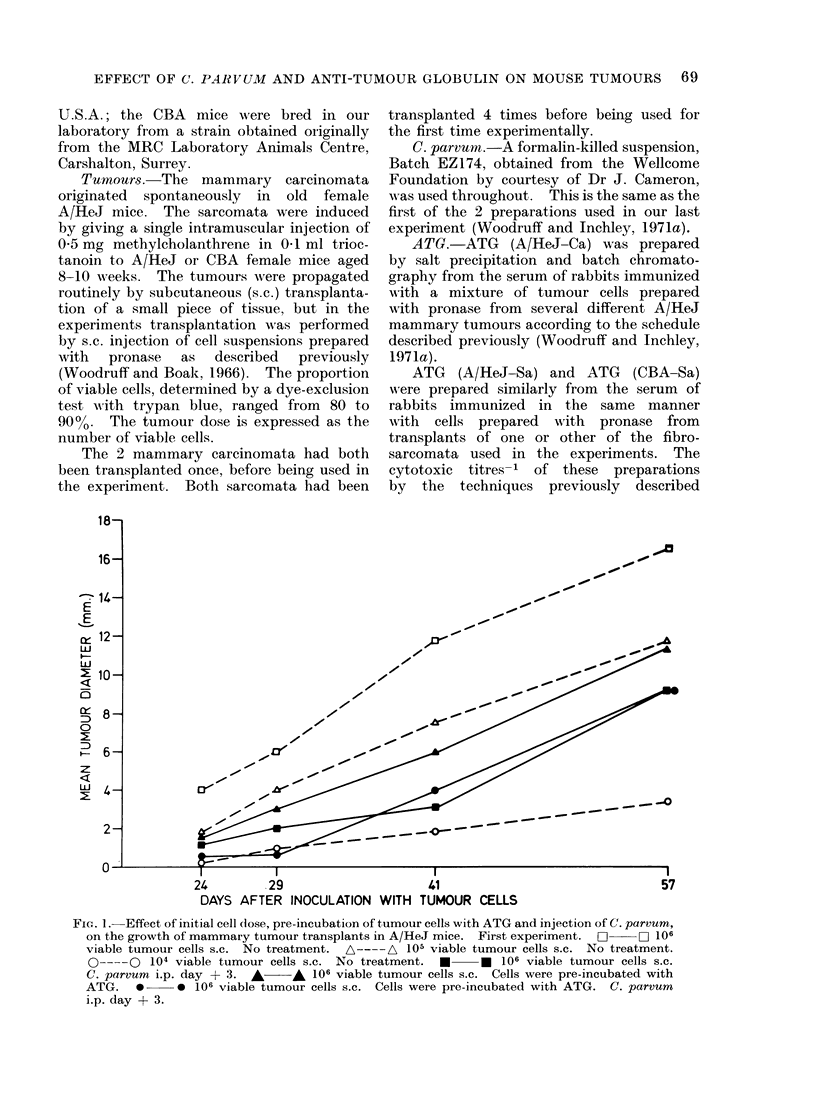

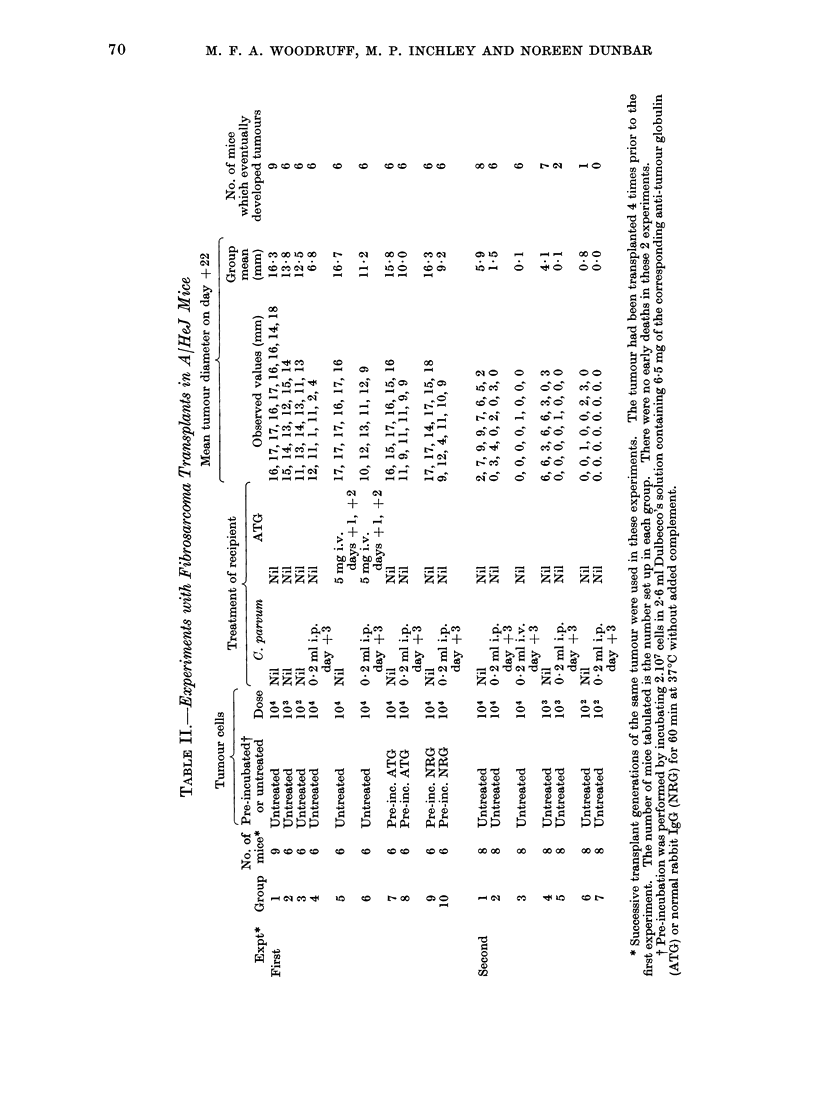

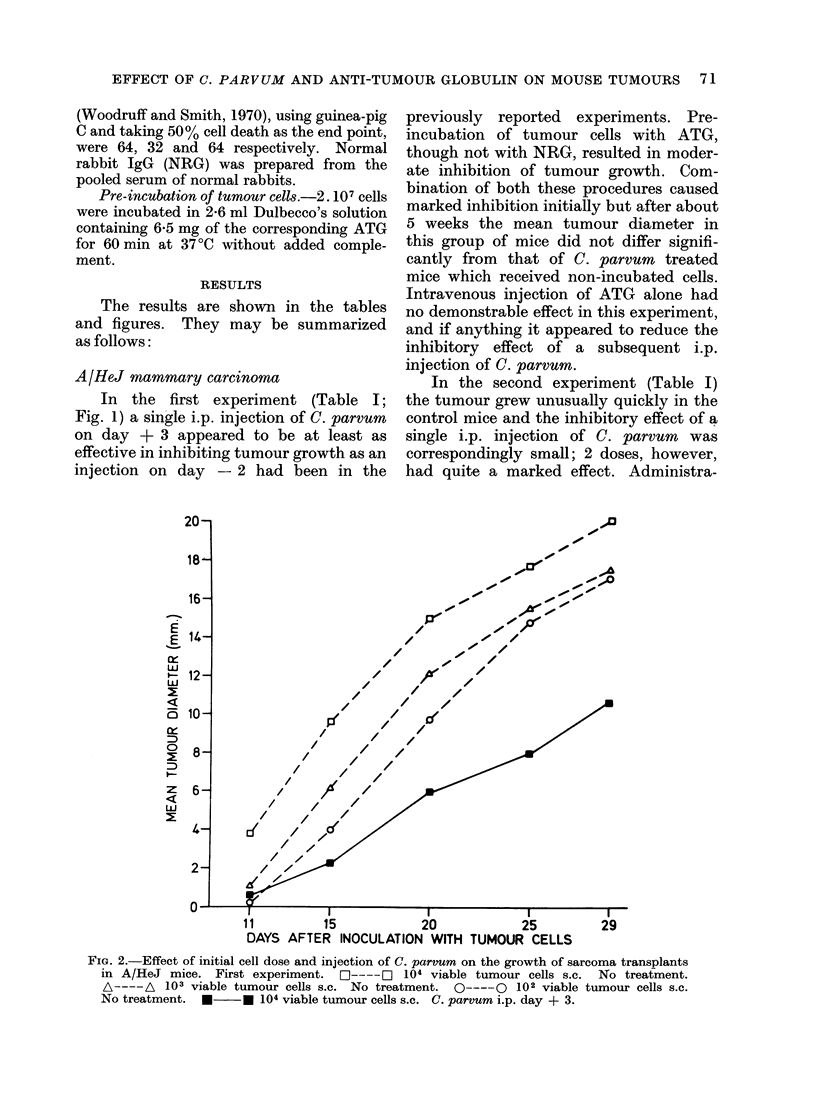

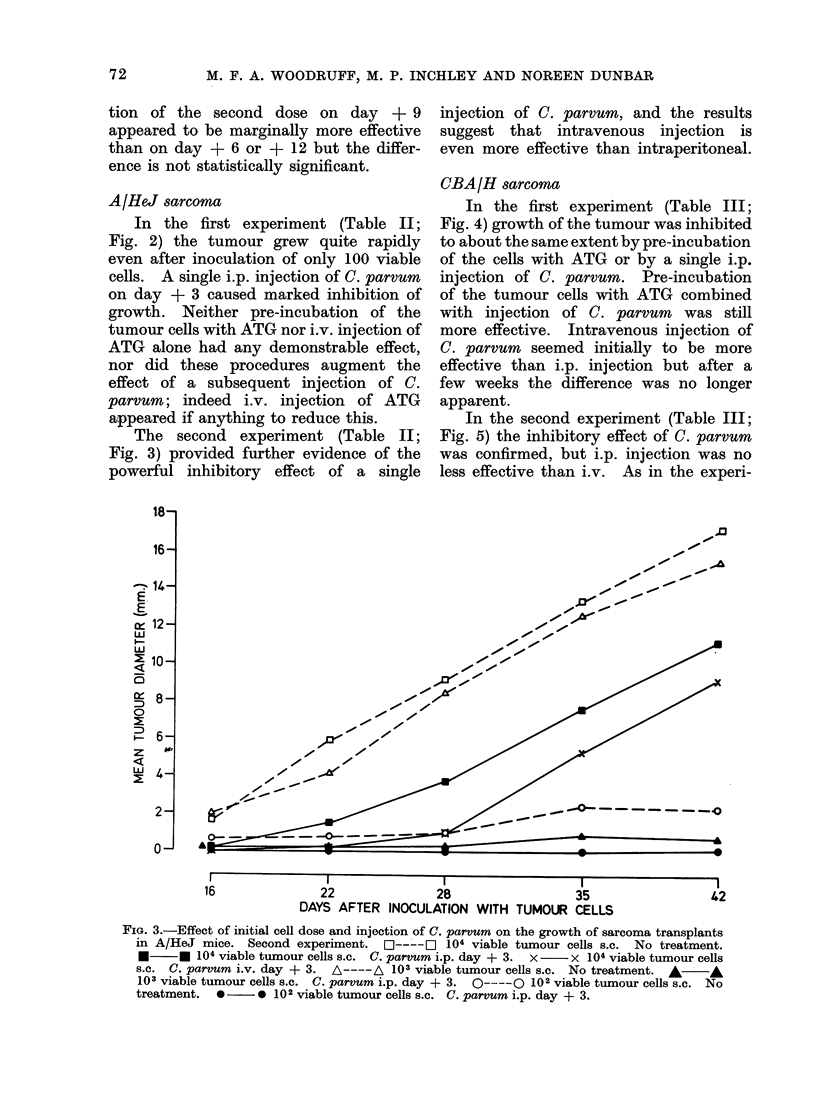

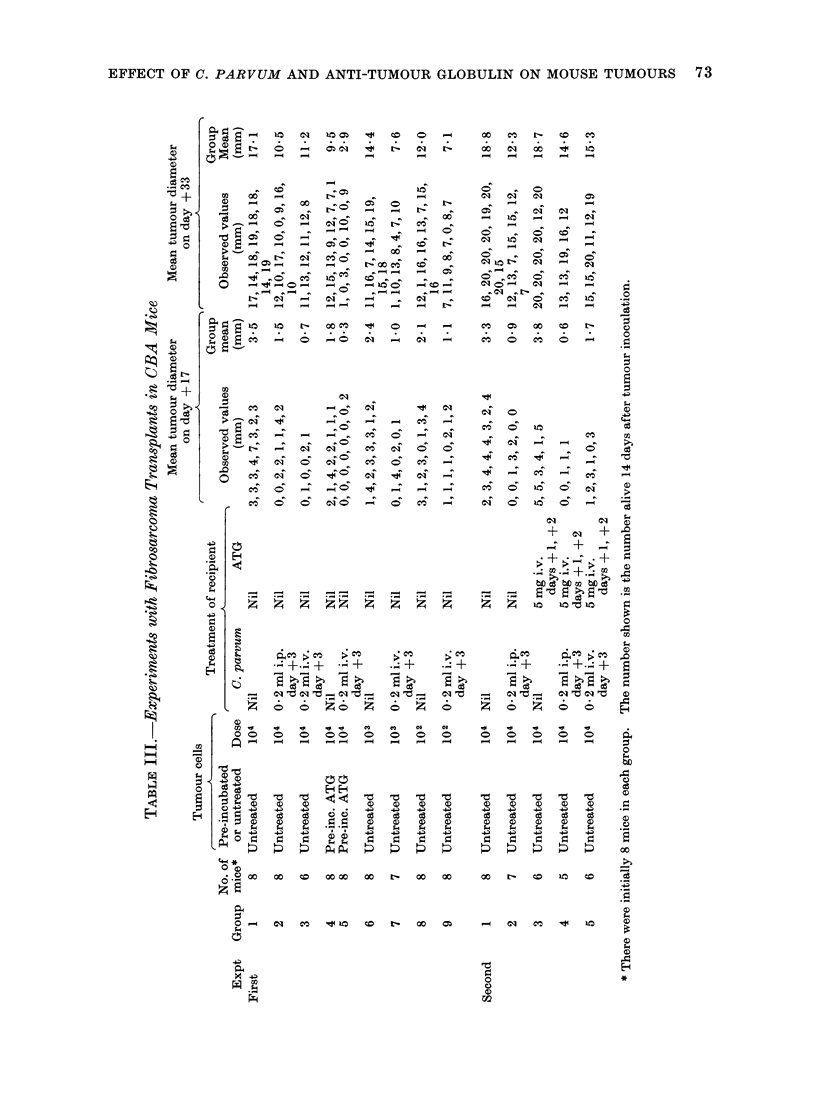

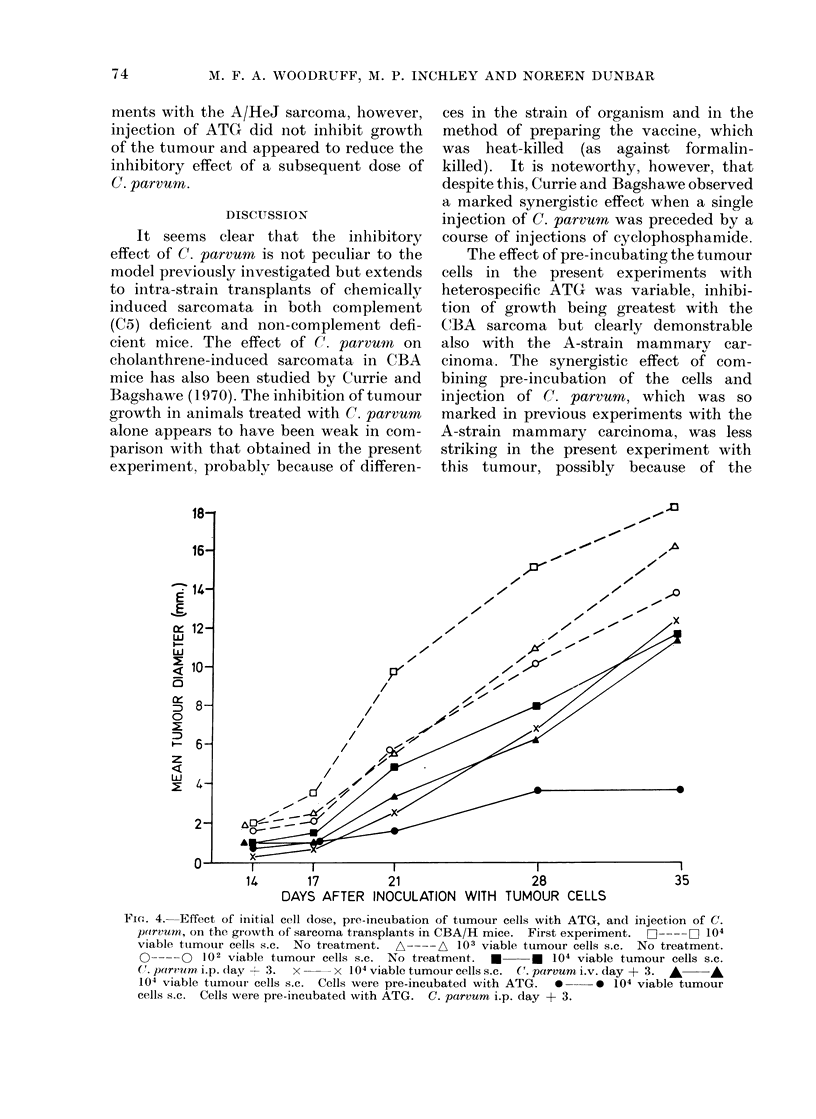

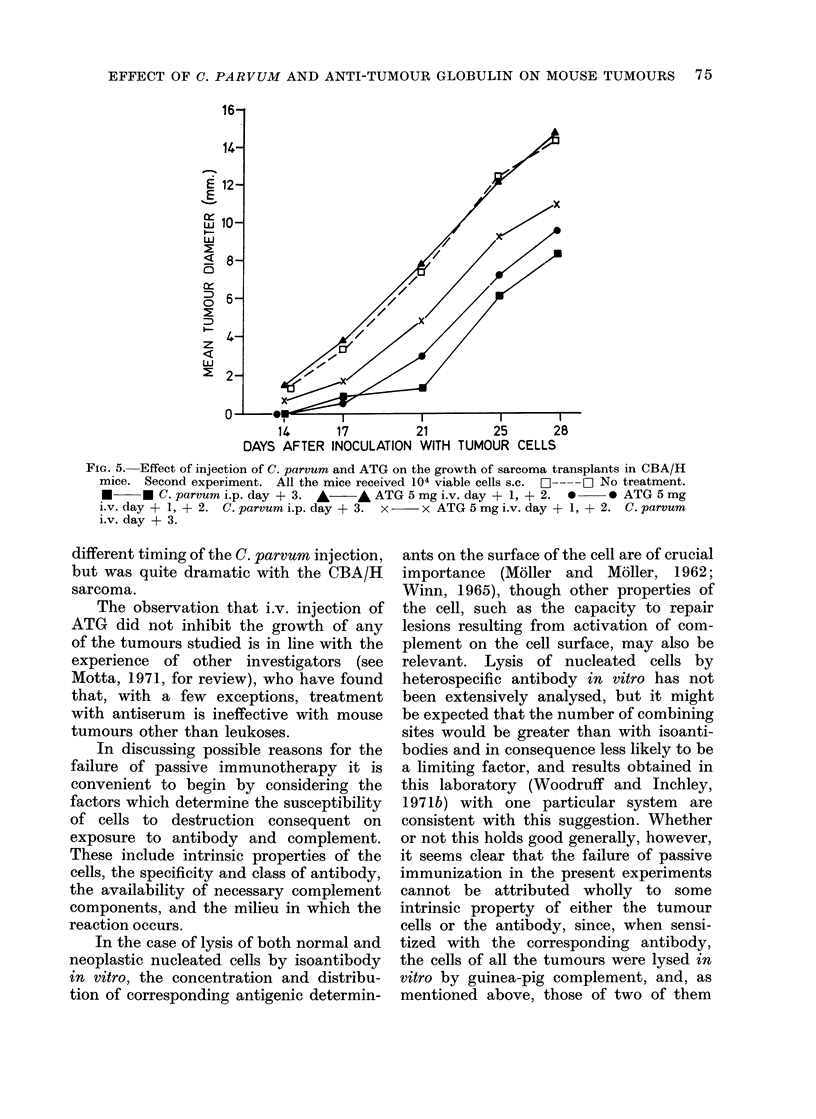

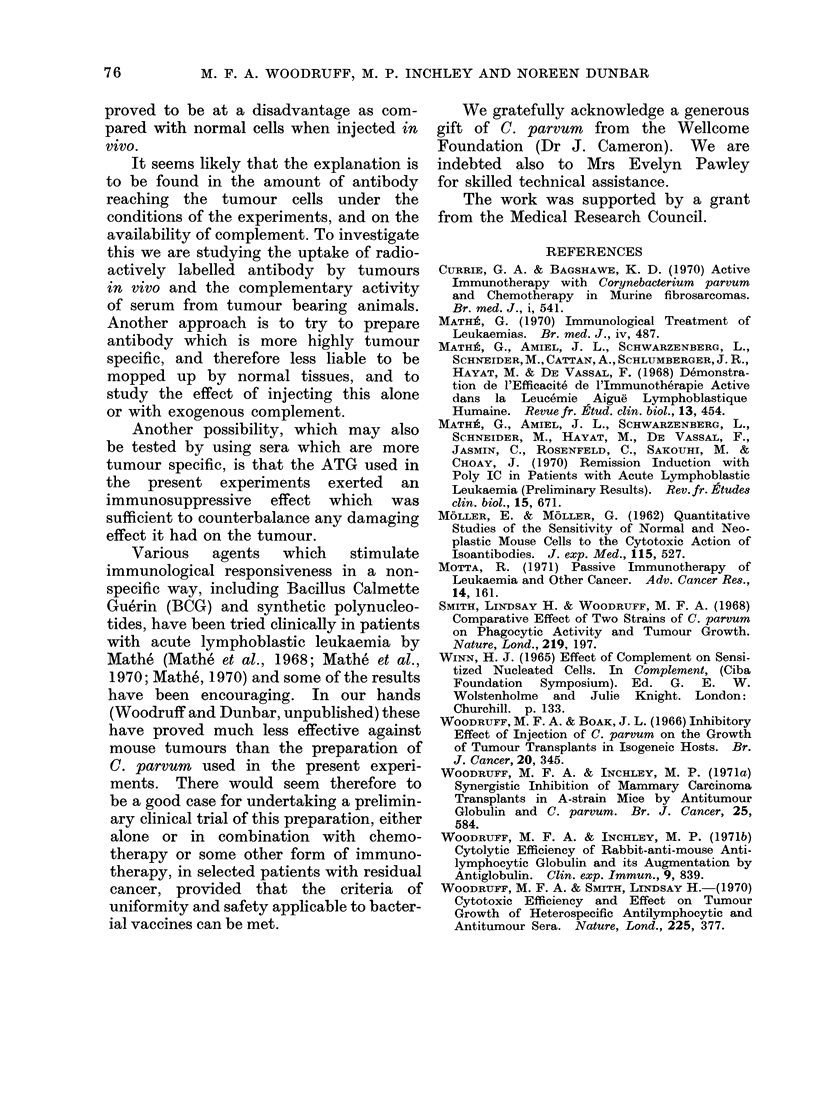

